# New horizons in prediction modelling using machine learning in older people’s healthcare research

**DOI:** 10.1093/ageing/afae201

**Published:** 2024-09-23

**Authors:** Daniel Stahl

**Affiliations:** Department of Biostatistics and Health Informatics, Institute of Psychiatry, Psychology & Neuroscience, King’s College London, London, UK

**Keywords:** prediction modelling, machine learning, precision medicine, older people

## Abstract

Machine learning (ML) and prediction modelling have become increasingly influential in healthcare, providing critical insights and supporting clinical decisions, particularly in the age of big data. This paper serves as an introductory guide for health researchers and readers interested in prediction modelling and explores how these technologies support clinical decisions, particularly with big data, and covers all aspects of the development, assessment and reporting of a model using ML. The paper starts with the importance of prediction modelling for precision medicine. It outlines different types of prediction and machine learning approaches, including supervised, unsupervised and semi-supervised learning, and provides an overview of popular algorithms for various outcomes and settings. It also introduces key theoretical ML concepts. The importance of data quality, preprocessing and unbiased model performance evaluation is highlighted. Concepts of apparent, internal and external validation will be introduced along with metrics for discrimination and calibration for different types of outcomes. Additionally, the paper addresses model interpretation, fairness and implementation in clinical practice. Finally, the paper provides recommendations for reporting and identifies common pitfalls in prediction modelling and machine learning. The aim of the paper is to help readers understand and critically evaluate research papers that present ML models and to serve as a first guide for developing, assessing and implementing their own.

## Key Points

- Precision medicine aims to improve healthcare by developing tailored prediction models using big data and machine learning.
- Choosing between machine learning and statistical models in healthcare depends on balancing adaptability and interpretability.
- Developing clinical prediction models involves a seven-step approach including development, evaluation, validation and presentation.
- Internal validation without data leakage is crucial to ensure unbiased estimates of performance.
- Involving all stakeholders enhances predictive model utility for patient care but raises compliance, cost and security concerns.

## Introduction

Evidence-based medicine (EBM) remains the dominant paradigm in medical practice: typically, a treatment is validated by randomised controlled trials and then applied across all applicable patients, even though not all may benefit from it. This standardised approach, designed for the ‘average person’, ensures adherence to proven methods but often fails to accommodate individual patient variability [1]. In recent years, a shift towards precision medicine has occurred. Unlike EBM, precision medicine assumes that reality is heterogeneous and that an average treatment response is not sufficient for all individuals. Precision medicine focuses on identifying which treatment (or other health-related approaches, such as preventive healthcare, diet or mental health care) will be effective for an individual based on a patient’s genetic, environmental and lifestyle factors [2]. Precision medicine has a long history, but it became only possible on a large scale in this century by the provision of ‘new’ data—such as high-resolution brain imaging, omics, electronic patient health records, sensors in wearables, smartphones and the internet—and high-performance computing technology which effectively collects, processes, stores and analyses the huge volume of available information which is commonly known as ‘Big data’ [3]. Prediction modelling and ML are essential for effectively developing personalised treatment plans, making them fundamental to the success of precision medicine.

Data-driven approaches, employing statistical and machine learning methods on often high-dimensional data (datasets with a very large number of features or variables compared to sample size), are used to improve disease classification, predict the risk of developing a disease, prognosticate the likely clinical course of a condition, predict treatment outcomes and optimise treatment selection [4, 5]. A recent study highlighted the importance of machine learning in three key areas of the healthcare of older people: first, in monitoring and early diagnosis through the detection of behavioural or physiological changes using wearable devices; second, in providing personalised care by tailored health and lifestyle advice, such as diet plans; and third, in improving health care coordination [6]. A review by [7] on machine learning applications in ageing and geriatric diseases found that the diagnosis and prediction of neurodegenerative disorders were the most researched areas. This was followed by noncommunicable diseases such as diabetes, hypertension, kidney and cardiovascular diseases and cancer, as well as mental health problems, particularly depression. They pointed out a lack in validating models across large and diverse datasets to ensure generalisability across age groups, genders, ethnicities and other demographics. This issue is common in many healthcare sectors and a primary reason why only a few models are routinely implemented in clinical practice [8].

This article introduces core concepts of developing clinical prediction using machine learning and statistical methods, essential for advancing precision medicine. It is aimed to help readers understand and critically evaluate research papers that use these models and to serve as a preliminary guide to developing, assessing and implementing their own. For an introduction to prognostic research using statistical modelling see [9], and for an introduction to ML, refer to [10].

### Fundamentals of prediction modelling

Clinical prediction models are becoming increasingly popular in health research though often with suboptimal methodology [11]. To address this, [12] proposed a seven-step approach for developing clinical prediction models, which involves addressing the (i) research question and initial data inspection, (ii) data preprocessing and predictor coding, (iii) model specification, (iv) model estimation, (v) model performance evaluation, (vi) internal (and external) validation and (vii) model presentation. Beyond Steyerberg and Verhoeven’s framework, assessing the fairness of the model [13], securing acceptance from clinicians, patients and other stakeholders and implementing it into the electronic health system for user-friendliness are additional important steps for a successful implementation in clinical practice [14].

### Defining a precise research question

Defining a precise research question in prediction modelling is crucial yet often overlooked. Is the aim to develop a prediction model that accurately predicts outcomes for new cases or is there also an aim to understand which factors are predictive and gain an understanding of our model? The exact aim will define the type of model to build. Often, the focus is on comparing numerous machine learning algorithms to identify the optimal model for the present data set. While it is important to demonstrate that ML models offer advantages over traditional modelling techniques, comparing too many models unintentionally risks overfitting, as it selects a model that may capitalise on random patterns in the specific dataset and may not generalise well [15]. Moreover, the quest for the ‘best’ model may overshadow the crucial understanding of predictive mechanisms and real-world applicability. Simple model comparisons should therefore not be the aim of a health research study. The research question will then determine the main outcome, such as predicting the progression from mild cognitive impairment to Alzheimer’s disease within 3 years postoperative delirium among older patients after major surgical procedures [16], estimating the time until the occurrence of all-cause mortality among older individuals [17] or identifying those at risk of frailty, as measured by a continuous frailty index, using electronic health records without requiring clinician oversight [18].

### Identifying variables and sample size considerations

After the research question has been formulated and the clinical outcome defined, existing knowledge on potential predictors needs to be reviewed, which may allow subject matter knowledge to be included in the design and development of the prediction model. A theory-driven variable selection is often advantageous because it uses established knowledge and insights from prior research or theoretical frameworks [19]. This ensures that the selected variables have a logical and theoretically justified relationship with the outcome, which helps prevent overfitting, enhances generalisability and improves interpretability. This is especially important for smaller data sets where there are limitations on the number of variables that can be included in a model. However, a theory-driven approach is often not feasible for big data sets with many variables such as in genomic studies or medical imaging.

Rules of thumb such as requiring at least 10 events (not cases) per variable lack reliability and recently sample size estimation for regression models have been established [20]. However, they have not yet been assessed or extended to more complex machine learning models. Recent simulations suggest that larger sample sizes are needed [21]. Often adaptive sample size determinations are used for more complex models. These are iterative approaches where the sample size of the dataset is dynamically adjusted based on the model’s performance during the development of the model [22].

### Data preprocessing

Data preprocessing is the first and often the most time-consuming phase in the development of a machine learning model, converting raw data into a format needed for modelling [23]. Data preprocessing involves several crucial steps to ensure the integrity and usefulness of the data. Assessing data quality is essential in data preprocessing, as the ‘garbage in, garbage out’ principle highlights that poor input data results in unreliable outputs [24]. Data preprocessing includes data integration, which involves merging data from different resources, resolving conflicts from different scales or encodings and data cleaning to correct inconsistencies and errors. Electronic health records (EHRs) often include unstructured clinical text, making data extraction a crucial step in data preprocessing. A machine learning method, Natural Language Processing, is used to extract information like symptoms, diagnoses, comorbidities, medications and potential side effects or treatments from unstructured text, transforming it into structured, interpretable data for analyses [25].

It is important to avoid basing variable selection solely on relationships between predictors and the outcome during the preprocessing phase, such as selecting only variables which significantly correlate with the outcome. This approach can lead to unstable and overfitted models [26]. Instead, variable should be selected independently of its relationship to the outcome or variable selection should be integrated in the model development process [27].

### Model specification

Next, a model needs to be specified to apply to the data set. If the sample size is limited, traditional regression models like linear, logistic or Cox regression are often appropriate. They may even outperform machine learning methods in low-dimensional settings where the number of features is small relative to the sample size or when the data contains significant noise or missing values [28]. The inclusion of fractional polynomial terms or restricted cubic splines further enhances the competitiveness of regressions against machine learning methods, as they provide a flexible way to model nonlinear relationships [26, 29].

If data sets are more complex, classical methods are often not appropriate anymore and machine learning and artificial intelligence (AI) methods need to be used. Machine learning (ML) methods offer advantages over traditional statistical modelling in their ability to handle complex high-dimensional data (such as genomics and omics, medical imaging, text, internet and sensor data) with unknown interactions and nonlinear relationships [30]. Unlike statistical modelling, which focuses on understanding and interpreting relationships with a predefined structure, machine learning learns directly from the data without explicit model specifications. This process involves computer-intensive optimisation of hyperparameters [31]. Hyperparameters are set manually and guide the learning process in machine learning algorithms, unlike model parameters, such as regression coefficients, which are estimated from data using methods like maximum likelihood. Hyperparameters influence how model parameters are estimated thereby impacting model performance and selection of the optimal model. Hyperparameter tuning involves adjusting the algorithm’s hyperparameters to optimise prediction accuracy on new, unseen data. The tuning process involves systematically searching for the hyperparameters that balance the model’s complexity, ensuring it is neither too complex, which could lead to overfitting, nor too simple, which might result in underfitting [10]. This balance is crucial for accurate predictions of new cases. The ideal parameters are usually determined through a method called cross-validation, detailed further in ‘Model Development and Assessment’.

Some ML algorithms, in particular Deep Learning, excel in automatic feature engineering and variable selection. They effectively capture complex patterns and relationships in diverse and unstructured data, adapt to new or changing data and maintain performance as data increases (scalability). Deep learning processes large datasets using a hierarchical architecture of algorithms known as neural networks, which mimic the human brain’s structure to identify complex patterns and relationships. This makes them particularly suitable for large datasets and unstructured data types like fMRI scans and other medical images, videos, speech, text or wearable sensors [32, 33]. For instance, deep learning has been used to analyse photographs of retinal images to identify individuals who may have Alzheimer’s disease. This technique has the potential to be implemented as a low-cost, noninvasive screening procedure within community eye-care infrastructures [34]. Large language models are deep learning tools trained on text data from sources like the internet or clinical health records. They are expected to significantly impact clinical care, research and medical education, but they require careful use because they can perpetuate existing biases and may generate false information [35].

### Types of machine learning algorithms

Machine learning sometimes uses a different terminology than statistical modelling. It is typically divided into supervised learning, unsupervised learning and reinforcement learning [36]. Supervised learning the algorithm learns from data (with labelled outcomes such as ‘healthy’ or ‘ill’) to predict outcomes for unseen cases. It is divided into regression (continuous outcomes, such as Hospital Anxiety and Depression Scale (HADS) score) and classification (categorical outcomes, such as ‘depressed’ or ‘not’) methods. In statistical modelling, models with categorical outcomes would also be named as regression models (i.e. logistic regression which models a continuous probability). Unsupervised learning involves identifying structure in data without labels (i.e. outcome), such as organising similar cases in groups (cluster analyses) or identifying how the data is distributed (density estimation). In prediction modelling, identified clusters are often used to predict an outcome. For example, [37] used hierarchical clustering on principal components in population-based survey data to identify a cluster of cases with a high likelihood of dementia. Reinforcement Learning is a machine learning approach where a computer agent (the algorithm) interacts with a dynamic environment to optimise decision-making, guided by feedback in the form of rewards and punishments. It is used in healthcare to optimise dynamic treatment regimens over time when decision-making is sequential and dynamic [38]. For instance, [39] developed a system that employs reinforcement learning to assist patients in following treatment plans by self-tuning to the patient’s skills and actions.

### Choice of model

The benefits of ML come with challenges such as overfitting, lack of generalisability to new settings and reduced interpretability. Overfitting occurs when a complex algorithm fits the training data too closely, resulting in poor generalisation to new data. Regularisation methods, which limit model complexity, and cross-validation, which assesses models on unseen cases, mitigate overfitting, while collecting representative data improves model performance and generalisability [10]. The interpretability of a model refers to how easily a human can understand the reasons behind a model’s prediction. This aspect is particularly important in health care, where understanding the decision-making process is crucial for trust among clinicians and patients, ethical responsibility and advancement of science [40]. Interpretable models are characterised by simple relationships between predictors and outcomes, such as easily interpretable parameters like regression coefficients and decision rules. Explainable AI aims to make the outputs of machine learning models more transparent and understandable, enhancing the ability of users to validate and trust AI systems [41].

The choice between ML and statistical approaches should be guided by the research question, data characteristics and analysis goals. Widespread are the so-called regularised regression models, such as Ridge, Lasso or Elastic Net, that bridge the gap between traditional statistical models and machine learning approaches [42]. They are popular because they offer a balance between the flexibility and adaptability of machine learning models and the robustness and interpretability of traditional statistical models. They are especially favoured in scenarios with high-dimensional datasets, correlated features or when automatic feature selection is needed. However, they may be less useful if data are complex with unknown interactions and nonlinearities, in which case other machine learning methods like random forest, neural networks or deep learning are preferable. Popular machine-learning algorithms are listed in Table 1. For a user-friendly introduction with software examples in R or Python, see [10, 30].

**Table 1:** Popular machine learning algorithms: learning type algorithm and basic working principle. For details see [10, 30]

Algorithm	Learning type	Basic working principle
Regularised Linear Regression	Supervised	Extension of the linear regression by including a regularisation penalty to prevent overfitting. Finds a linear relationship between input variables and a continuous output variable
Regularised Logistic Regression	Supervised	Extension of the logistic regression by including a regularisation penalty to prevent overfitting. Estimates probabilities using a logistic function, often used for binary classification
Regularised Cox Proportional Hazards Model	Supervised	Extension of the Cox regression by including a regularisation penalty term. Used in survival analysis to model the time until an event occurs focusing on the relationship between survival time and one or more predictors and including censored data. Many machine learning algorithms have a version which allows modelling time until an event occurs
Decision Trees	Supervised	Splits data into branches to form a tree structure, making decisions based on features
Random Forest	Supervised	Ensemble of Decision Trees, used for classification and regression, improving accuracy and reducing overfitting
XGBoost	Supervised	A highly optimised machine learning library known for its speed and performance. It combines decision trees (‘weak learners’) sequentially to develop stronger learners to improve predictions. This is done by training each weak learner on the errors of the preceding one, targeting areas of poor model performance
Support Vector Machines	Supervised	An algorithm that helps to separate data points into distinct categories by finding the best-dividing line (or plane in more complex multidimensional situations) between different sets of data points
K-Nearest Neighbours	Supervised	Classifies data based on the majority vote of its ‘*k*’ nearest neighbours in the feature space
K-Means Clustering	Unsupervised	Partitions data into ‘*k*’ distinct clusters based on feature similarity
Hierarchical Clustering	Unsupervised	Creates a tree of clusters by iteratively grouping data points based on their similarity
Self-Organising Maps	Unsupervised	Neural network based, used for dimensionality reduction and visualisation, organising high-dimensional data into a low-dimensional map
Principal Component Analysis	Unsupervised	Reduces data dimensionality by transforming to a new set of variables (principal components)
Neural Networks	Supervised/ Unsupervised	Composed of interconnected nodes or neurons, mimicking the human brain, used in complex pattern recognition
Deep Learning	Supervised/ Unsupervised/ Reinforcement	Utilises multilayered neural networks to analyse large and complex datasets, excelling in complex tasks like image and speech recognition and natural language processing. Usually not recommended for small data sets
Q-Learning	Reinforcement	A model-free reinforcement learning algorithm that seeks to learn a policy, which tells an agent what action to take under what circumstances

### Model development and assessment

**Figure 1:**
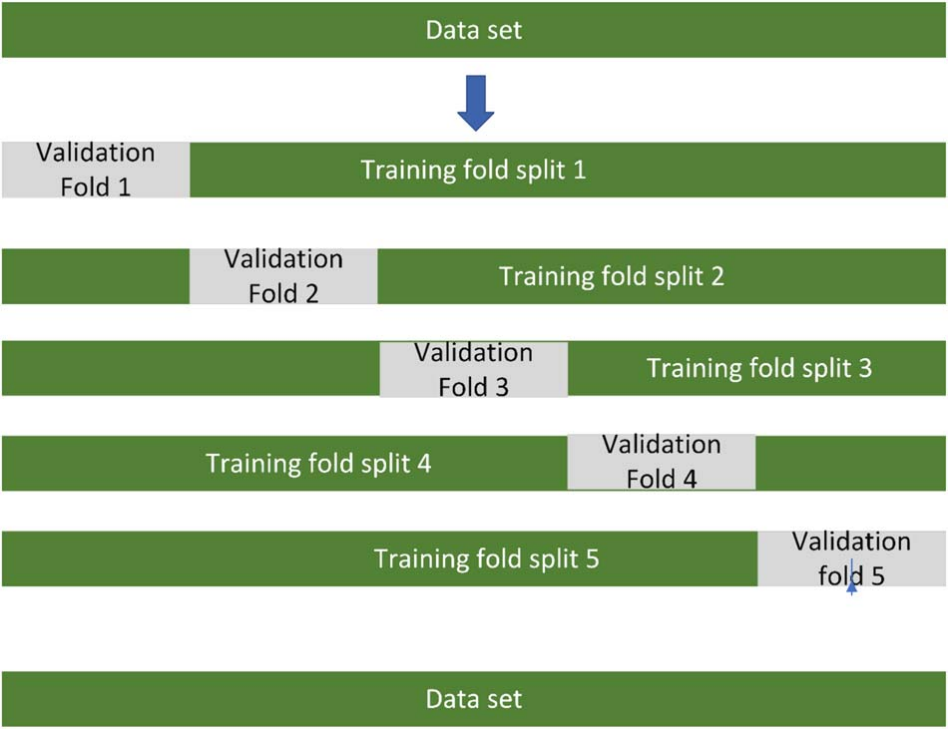
Hyperparameter tuning is a crucial step in optimising machine learning model performance. It involves finding the best configuration options (hyperparameters) that minimise prediction error. Typically, 5–10-fold cross-validation is used for this purpose. In 5-fold cross-validation, one-fifth of the data serves as an independent validation set, while the rest is used for training. The model is trained using different hyperparameter settings, and its performance is evaluated on the validation set with unseen data. This process is repeated five times, using each subset as the validation set once. After each iteration, the model’s performance metrics are recorded. The hyperparameters yielding the best overall performance, as determined by the averaged results across the 5 iterations, are selected as the optimal configuration. Finally, the model is trained using these chosen hyperparameters on the entire dataset for improved performance.

After identifying a suitable statistical or machine learning method, one must develop a model by fitting the data to it. If statistical modelling techniques are applied, the model is fitted to the entire data set and parameters are estimated using typically maximum likelihood methods as in standard statistical modelling analysis. However, if regularised regression or machine learning methods that require hyperparameter tuning are used, implementing cross-validation or bootstrapping procedures becomes necessary. These techniques help to identify the optimal set of hyperparameters which are usually those that optimise and minimise prediction error in unseen (hold-out) data (see Figure 1). The best set of hyperparameters is then used to fit the final model to the entire data set.

### Model performance assessment

Model performance assessment is typically conducted by evaluating three main aspects: overall model performance, discrimination and calibration [43]. Overall model performance quantifies how closely predictions match the actual outcomes. This can be measured using metrics like the explained variance *R*^2^ for continuous outcomes and the Brier score for binary outcomes. The Brier score is the average difference between the actual outcome and predicted probability and ranges from 0 (perfect accuracy) and 1 (perfect inaccuracy), with the cut-off for a noninformative model depending on the prevalence. Discrimination, the model’s ability to effectively distinguish between outcome levels (such as case and control), is measured by the *c* statistic for categorical outcomes [equivalent to area under the receiver operating characteristic curve (AUROC) for binary outcomes], with values ranging from 0.5 (no discrimination) to 1.0 (perfect discrimination). A value of 0.8 is often regarded as a very good discriminative ability, but performance should not be seen outside the context of the purpose of the model. For instance, a screening tool for common diseases may still be useful with an area under the curve (AUC) of <0.8 if the treatments are cost-effective and have no side effects. However, a high AUROC does not necessarily imply accurate predictions, which is where calibration comes in. Calibration relates the agreement between observed outcomes and predictions identifying the risk estimate ranges where the model performs well [44]. A well-calibrated model with binary outcomes will have predicted probabilities that align closely with observed rates. For example, if a model predicts a 70% chance of an event, we expect to see that event occurring ~70% of the time in patients with such a predicted probability. Calibration is often quantified by the calibration slope (ideal value of 1) and intercept (ideal value of 0) to evaluate prediction accuracy and bias. In addition, calibration plots are often used to visually inspect this agreement across predicted probabilities.

If a prediction model is used to guide treatment decisions, a cut-off to categorise patients into low-risk (i.e. no treatment required) or high-risk (i.e. treatment needed) groups may be needed. This cut-off should be determined by clinical expertise and by balancing costs and benefits as performance metrics like sensitivity, specificity, positive predictive value and negative predictive are threshold dependent and vary with the chosen cut-off [45]. Relying on a default 50% cut-off is often inappropriate. Patient perspectives should also be considered, as different individuals may accept varying levels of risk. Table 2 provides an overview of commonly used performance metrics for categorical outcomes.

**Table 2:** Key metrics for evaluating prediction models with categorical outcomes—definitions, formulas and interpretations

Term	Explanation	Formula
Sensitivity (recall)	The ability of a test to correctly identify those with the condition (true positive rate)	Sensitivity = true positives/(true positives + false negatives)
Specificity	The ability of a test to correctly identify those without the condition (true negative rate)	Specificity = true negatives/(true negatives + false positives)
Negative predictive value (NPV)	The proportion of negative test results that are true negatives	NPV = true negatives/(true negatives + false negatives)
Positive predictive value (PPV) or precision	The proportion of positive test results that are true positives	PPV = true positives/(true positives + false positives)
F1 score	The F1 score is the harmonic mean of precision and recall, accounting both for false positives and false negatives	F1 score = 2 × (precision × recall)/(precision + recall)
Discrimination	The model’s ability to distinguish between different levels of outcome, often measured by metrics like the AUC for binary outcomes	AUC (area under the curve or area under the receiver operating characteristic curve) is not derived from a simple formula. It involves plotting true positive rate against false positive rate at various threshold settings and measuring the area under this curve. The AUC can be interpreted as the probability that the model ranks a random positive example higher than a random negative example
Calibration	The agreement between observed outcomes and predictions is assessed by calibration slope (beta) and intercept (alpha)	Calibration slope and intercept based on a logistic regression for categorical outcomes; often visualised with calibration plots, where the predicted probabilities are plotted on the *x*-axis and the observed frequencies on the *y*-axis. A perfectly calibrated model would result in a plot where the points lie on the diagonal line. In the ideal case, the calibration slope would be 1 and the intercept 0

Providing probabilities may often be more appropriate as it provides a spectrum of risk levels instead of a binary classification allowing for providing more personalised treatment based on the specific individual risk profile. Finally, clinical decision analyses like net benefit analysis are recommended. These methods assess the trade-offs between the benefits and risks of treatments with different treatment options to identify the treatment that provides the maximum benefit for the least cost and risk [46].

### Internal and external validation

Model performance estimates obtained using the same data as for model development (apparent validation) tend to be overly optimistic [47]. Such estimates are often of limited use, especially when the sample size is not sufficiently large. After developing a machine learning model, performance needs to be assessed on new data from the same underlying population (internal validation). Internal validation is the minimum requirement for assessing the performance of the machine learning model. Subsequently, the model should be evaluated on new datasets from different clinical or geographical settings, time periods or populations (external validation). For the final stage of external validation, particularly in a healthcare context, assessing the model’s acceptance and effectiveness through a clinical trial is typically needed before an implementation in the clinical practice can be considered.

### Additional considerations for validation

**Figure 2:**
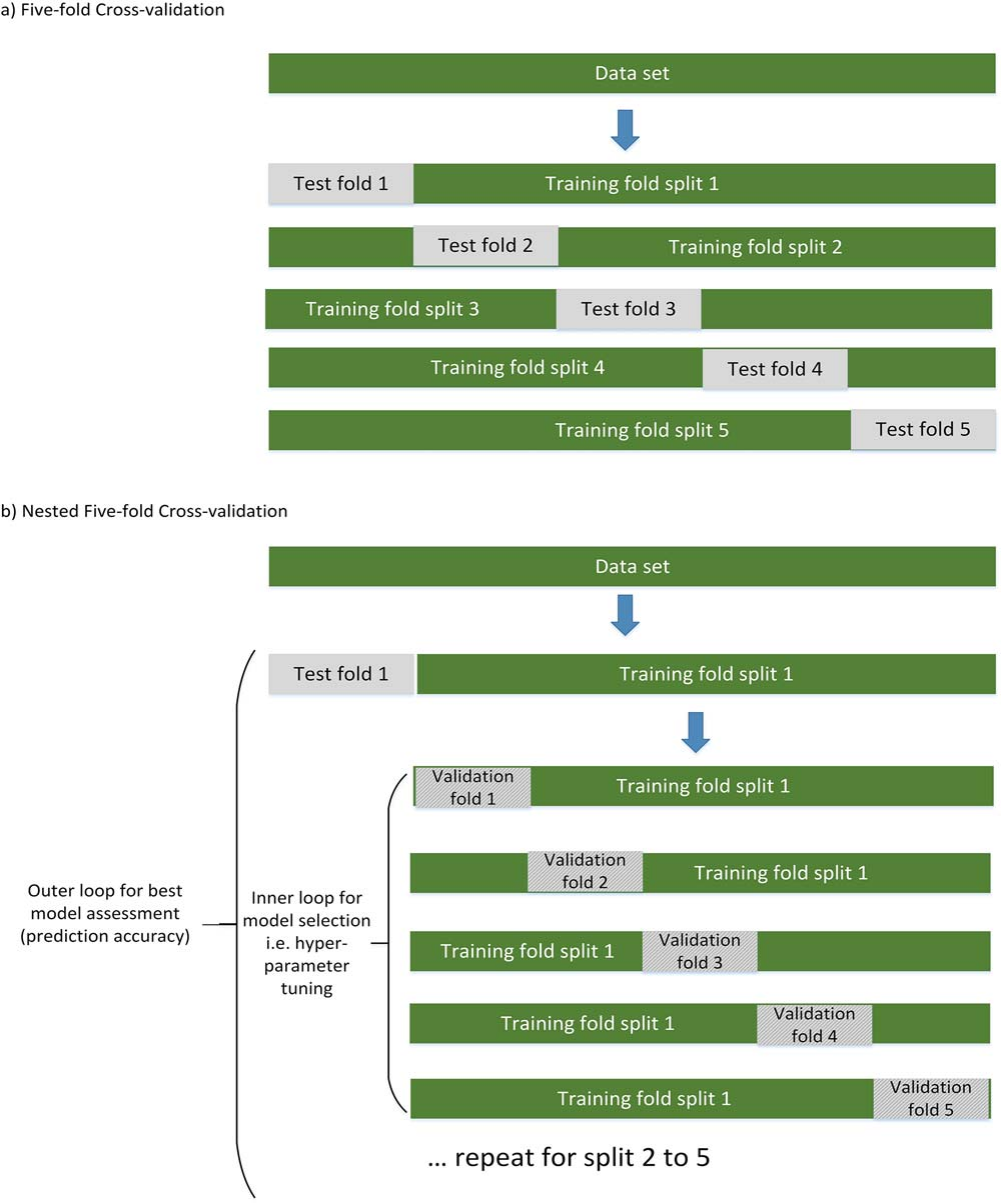
Model selection and model assessment using nested cross-validation. Model assessment without model selection, i.e. without hyperparameter selection as in regression models, can be performed using 5–10-fold cross-validation. Five-fold cross-validation, as shown in (a), retains one-fifth of the data as an independent test set for model assessment and the remaining four-fifths for training. This process is repeated across five data splits, with each case being used once as part of a validation dataset. The results of the five test folds are averaged, and the final model is fitted using the entire sample. If model selection, such as hyperparameter tuning, is undertaken, nested cross-validation (b) must be performed. Here, for each split of the data, an additional five-fold splitting of the training data is implemented. The inner loop is used for model selection and the outer loop for model testing. This arrangement of an ‘inner loop’ for model selection (hyperparameter tuning) and an ‘outer loop’ for model testing effectively prevents information leakage and ensures a more robust evaluation of the model’s performance. Additionally, to obtain a more stable estimate of performance, both the standard and nested cross-validation procedures can be repeated multiple times with different partitions of the data, and the results averaged across different these repeats.

It is important to recognise that model development and model assessment serve two distinct goals and should not be conducted on the same data. Independent datasets are necessary for each of the two tasks to prevent biassed or over-optimistic assessment of the model’s performance [42, 48]. This issue, commonly referred to as ‘data leakage’, occurs when external information is used during model development and still persists in the machine learning community [49]. To obtain a valid (unbiased) estimate of internal validity when developing machine learning models with hyperparameter selection, nested cross-validation is commonly used. Nested cross-validation involves two layers of cross-validation: the inner loop for hyperparameter tuning and the outer loop for model assessment; see Figure 2 for details. This separation of tuning and model assessment is crucial as it prevents information leakage from the test data into the model training process, thereby ensuring that the evaluation of the model’s performance remains unbiased.

It is important to note that the hyperparameters selected in the inner loop are not used for the final model estimation. Instead, the inner loop serves to mimic the process of model development to ensure a reliable estimate of model performance obtained from the nested cross-validation. Valid alternatives to nested cross-validation include bootstrapping procedures, such as optimism correction [26]. A split sampling approach (i.e. using 70% for model development and 30% for assessment) is not recommended as it can be unstable and inefficient, especially if the sample size is <20 000 [50]. An interesting approach to consider is the ‘internal-external’ validation procedure where data are not randomly split into *k* folds in the outer loop but are split by study site, hospital, catchment area or even calendar time. This approach allows for some form of external validation of the model [47].

### Missing data

Handling missing data is a common challenge in machine learning and often poorly handled [51]. Missing values are typically replaced with plausible values through imputation. In statistical modelling, multiple imputation, which involves creating multiple complete data sets with imputations based on a distribution of plausible values to reflect uncertainty, is the standard approach [52]. However, in machine learning, multiple imputations may not be the best approach [53]. Instead, data are often imputed using single imputation methods such as *k*-nearest neighbours, which impute values based on the similarity of cases with non-missing data, or by more complex methods such as random forest. Simpler methods like mean imputation should be avoided due to information loss and potential performance decline. It is crucial to prevent information leakage e.g. by imputing separately in training and test/validation sets [54] or by using the imputation model of the training set to predict missing data in the validation/tests sets [27]. Some algorithms such as random forest, XGBoost or neural networks can inherently handle missing data, eliminating the need for imputation preprocessing steps.

### Fairness and bias

Machine learning models are often used on the promise of increased objectivity in decision-making but algorithms are often not ‘fair’ [55] An algorithm is considered fair if it performs equally well across different groups of people without bias related to ethnicity, gender, sexual orientation, disability, age or social class. However, bias identification and mitigation attempts mainly focused on identifying gender and racial disparities while other demographic-related biases, such as age, have often been neglected [56]. Therefore, assessing the performance of clinical models for different groups is a crucial aspect of model evaluation.

### Reporting and presentation of the model

It is crucial to assess a model that the study’s reporting is open, reproducible and transparent. [57]. Adequate reporting allows a critical evaluation of a model. Reporting guidelines such as the Transparent Reporting of a Multivariable Prediction Model for Individual Prognosis or Diagnosis (TRIPOD) statement [58] provide guidelines and checklists on how to effectively present the development and validation of clinical prediction models [59] and AI models effectively [60]. Guidelines for developing and reporting for machine learning models provided by [61, 62] offer a guide for presenting clinical prediction models in a way that facilitates external validation and use by clinicians. The implications of the model for clinical use of the model, limitations and future research should be discussed [63]. Finally, making code and, when possible, data publicly available should be encouraged for potential analyses assessment.

### Implementation

The ultimate goal of machine learning models is their implementation in clinical settings to improve patient outcomes, a process that is challenging and time consuming [64]. Perhaps the most effective method is to embed them as electronic clinical decision support systems within the electronic health record system. This integration allows the automatic extraction of necessary clinical information and provides real-time insights for informed clinical decision-making [65]. Consequently, addressing the value of clinical decision judgement and detailing the pathway towards implementation should be an integral part of the model’s design and be discussed in any report [66].

The QRISK model illustrates the successful development and implementation of a clinical prediction tool using electronic health records to assess the 10-year risk of developing cardiovascular disease. The model was developed using a Cox proportional hazards model with fractional polynomials and achieved very good discrimination and good calibration in internal and external validation. Introduced in 2007, QRISK2 can be used by analysing data already available in a person’s EHR (https://www.nice.org.uk/guidance/ng238). The most recent version, QRISK3, performs well across the general population, but a recent study suggests only moderate discrimination among older individuals >65 years [67]. It tends to overestimate their risk which may lead to unnecessary medical treatments and stress for patients.

However, challenges such as integration with existing systems, regulatory and compliance regulations, costs and long-term maintenance must be considered. Additionally, privacy and data security concerns, potential biases along with a lack of trust from patients and healthcare providers in the validity and effectiveness of the model’s decision-making process present further barriers [68, 69]. Important is the involvement of patients which is still often overlooked or undervalued by data scientists potentially impacting the effectiveness and acceptability of the resulting models [70].

### Final words

Successfully addressing the challenges of developing and implementing ML models in clinical practice requires a collaborative effort from all stakeholders and a multidisciplinary approach. Involving clinicians, data scientists, researchers, policymakers and patients in the development, validation and implementation stages can lead to more robust and reliable ML models. These models can then serve as successful clinical decision tools to support evidence-based and personalised decision-making, ultimately improving patient care and outcomes. This paper aims to help clinicians and healthcare researchers assess the methodological quality and clinical potential of models, whether reviewing studies or conducting their research. To assist readers, Table 3 provides key questions to evaluate whether a prediction model meets essential clinical standards.

**Table 3:** Evaluation criteria questions for prediction modelling and machine learning research studies

Evaluation aspects	Key questions for assessment
Research question	Is there a clear research question or objective with an adequate definition of the outcome?
Study design and population	Are study design and characteristics of the population adequately described, including the sample size and any inclusion or exclusion criteria?
Data collection and preprocessing	Are details about the data collection process, including the variables collected and any preprocessing steps provided? Are all variables well defined? Are no uni- or multivariate methods used to preselect variables based on association with the outcome?
Variable selection and model development	Are the methods used for variable selection and model development described? Is the modelling method, including model tuning or optimisation procedures, explained? Which method was used for optimisation to avoid overfitting (i.e. cross-validation or bootstrapping)? Does model development and performance estimation avoid data leakage?
Missing data	Are missing data adequately reported and are cases with missing data compared on basic sociodemographic characteristics with complete cases? Are missing data handling procedures reported (complete case analyses, imputation methods) and justified? Is data leakage avoided?
Model performance evaluation	As a minimum, internal validation should be done for performance assessment! Are adequate measures for discrimination and calibration reported? Are threshold cut-offs for measures such as sensitivity and specificity provided? Did they assess clinical usefulness? If hyperparameter tuning was performed: Did they perform nested cross-validation or similar procedures to get an unbiased estimate of prediction accuracy? Did the author avoid any form of data leakage? Did the authors compare accuracy across age, gender and ethnicity groups to ensure fairness across diverse health populations?
Model reporting	Is the final model presented (i.e. regression formula) or available as a web tool? Is the model transparent about the model’s inputs, outputs and underlying assumptions and discusses how easily interpretable the model is for clinicians or end-users? Did they assess potential biases by comparing prediction accuracy between different sociodemographic groups?
Discussion	Does the discussion section provide a thorough interpretation of the results, including the implications and limitations of the findings? Is there a discussion of the generalisability of the model and its potential impact on clinical practice? Do they discuss the next steps towards implementation?
Limitations and potential biases	Are limitations of the study, including potential sources of bias or uncertainty in the data or modelling process, discussed?
Open science	Are the data and code used for model development and assessment available and accessible to the scientific community?
